# Serpin7 controls egg diapause of migratory locust (*Locusta migratoria*) by regulating polyphenol oxidase

**DOI:** 10.1002/2211-5463.12825

**Published:** 2020-03-24

**Authors:** Jun Chen, Dongnan Cui, Hidayat Ullah, Kun Hao, Xiongbing Tu, Zehua Zhang

**Affiliations:** ^1^ State Key Laboratory for Biology of Plant Diseases and Insect Pests Institute of Plant Protection Chinese Academy of Agricultural Sciences Beijing China; ^2^ Department of Agriculture The University of Swabi Pakistan

**Keywords:** diapause, locust, *Locusta migratoria*, polyphenol oxidase, RNAi, serpin

## Abstract

Diapause is a state of arrested growth, which allows insects to adapt to diverse environments. Serine protease inhibitors (serpins) play an important role in various physiological processes, including blood coagulation, fibrinolysis, development, complement activation and extracellular matrix remodeling. We hypothesized that serpin may affect energy metabolism and thereby control diapause of migratory locust (*Locusta migratoria*) embryos by regulating protease cascades. A total of seven nonredundant serpin genes (named *serpin1*–*serpin7*) of *L. migratoria* were obtained through transcriptomic analysis. We further performed label‐free proteomic sequencing and analysis of diapause and nondiapause eggs of *L. migratoria*, revealing significant differences in *serpin7* expression. A significant reduction in diapause rate under the short photoperiod was observed in insects treated with *serpin7* double‐stranded RNA. Furthermore, knockdown of the *serpin7* gene resulted in significant upregulation of the activity of polyphenol oxidase. We therefore propose that the observed *serpin7* gene plays a crucial role in diapause, suggesting that control of energy metabolism may have potential as a future strategy for the reproductive control of insect pests.

## Graphical abstract

Lm_Serpin7, a member of the Serpin protease inhibitor family, is highly expressed in diapause eggs and plays an important role in *Locusta migratoria* egg diapause. Knockdown of Lm_Serpin7 by injection with ds_*serpin7* decreases egg diapause rate, suggesting that control of Lm_Serpin7 may have potential as a future strategy for the reproductive control of insect pests.

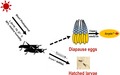

**Abbreviations**
*A*: absorbance
ddH_2_O: double‐distilled water
dsRNA: double‐stranded RNA
LB: lysogeny broth
NJ: neighbor‐joining
ORF: open reading frame
pI: isoelectric point
PO: phenol oxidase
PPO: polyphenol oxidase
proPO: prophenoloxidase
RH: relative humidity
RNAi: RNA interference
RT‐qPCR: real‐time quantitative PCR

Diapause is an important behavior of insects that allows the insects to adapt to diversified environments. Migratory locust, *Locusta migratoria* L., belongs to the facultative diapause type of embryonic diapause insect [1, 2]. Temperature and photoperiod are the key factors affecting diapause of *L. migratoria*. The maternal *L. migratoria* can sense the environmental information and transmit this information in the form of signals to the offspring to cut off the developmental signal, which induces diapause of the eggs in the locusts [3].

Serine protease inhibitors (serpins) belong to the widely distributed protein family protease inhibitors [4, 5]. More than 1500 serpins have been extensively studied in animals, plants, bacteria and viruses [6]. Generally, the serpin could be 350–500 amino acids found in typical serpin, and irreversible inhibition occurs whenever serpin binds to its substrates [4, 7]. Studies showed that the serpins play an important role in blood coagulation, fibrinolysis, complement activation, inflammatory reactions, immunity, physiology, digestion, development and extracellular matrix remodeling [8, 9, 10, 11, 12]. Seven serpins were purified from hemolymph of tobacco moth, *Ephestia* *elutella*, that regulated the activation of phenol oxidase (PO) and played a role in immune defense [13, 14, 15]. In addition, serpins also play a crucial role in tissue synthesis and embryonic development of animals [16]. *Spn27A* regulates the formation of dorsal ventral axis of drosophila embryos in the early developmental stage by inhibiting the Toll signaling pathway [17, 18]. In addition, *Spn88Ea* is necessary for wing development in fruit flies [19]. Balance of *SRP‐2* (serpin), a cross‐class inhibitor, is also important for postembryonic development of nematodes, *Caenorhabditis elegans* [20]. However, no extensive and comprehensive studies have been made on the effects of serpin in relation with insect diapause. Keeping in view the importance of serpins in other plants and animals, this novel study was designed to carry out the transcriptome analysis of diapause and nondiapause eggs of migratory locust, *L. migratoria*, especially for the serpin genes [21, 22]. We further performed label‐free proteomic sequencing on diapause and nondiapause eggs of migratory locust to understand the expression of serpin‐related genes [21, 22]. We hypothesized that serpin may affect energy metabolism and could control diapause of migratory locust embryos by regulating protease cascade reaction. To explore the role of serpin in diapause regulation, we performed RNA interference (RNAi)‐mediated silencing of serpin gene. We further planned to study the content of polyphenol oxidase (PPO) by applying RNAi of specific serpin gene. This study would provide a theoretical basis for further study on the diapause mechanism of the migratory locust through serpins.

## Materials and methods

### Insect materials

The *L. migratoria* L. colony used in this study was originally collected from the field at Cangzhou, Hebei, China (39°37′N, 98°30′E, 40 m above sea level) and was maintained by the State Key Laboratory for Biology of Plant Diseases and Insect Pests, Institute of Plant Protection, Chines Academy of Agricultural Sciences for successive years. Locust eggs were hatched in an artificial climate box (PRX‐250B‐30; Haishu Saifu Experimental Instrument Factory, Ningbo, China) at a temperature of around 30 °C with relative humidity of 60%. The photoperiodic regimen used for nondiapause locusts in the experiment was 16 h light : 8 h darkness. Similarly, to induce diapause, we reared locusts under a short photoperiod at 10 h light : 14 h darkness, 27 °C and 60% relative humidity [23, 24]. Freshly grown wheat seedlings were fed to the locusts in the laboratory.

### Identification of serpin genes in the migratory locust

The transcriptome sequencing and analysis were performed on diapause and nondiapause eggs of migratory locusts in the State Key Laboratory for Biology of Plant Diseases and Insect Pests, Institute of Plant Protection, Chines Academy of Agricultural Sciences. A total of seven serpin genes (*serpin1* to *serpin7*) were obtained. Molecular formula, molecular weight and isoelectric points of serpin proteins were analyzed by expasy software (Swiss Institute of Bioinformatics, Geneva, Switzerland). Meanwhile, we used wolf psort software (http://wolfpsort.org/) to predict the subcellular localization of serpins (Data S1).

### Amino acid sequence alignment and construction of phylogenetic tree

dnaman software (version 7.212; Lynnon Corp., Quebec, QC, Canada) was used to translate the serpin sequence of migratory locust. We obtained the amino acid sequences of serpins’ open reading frame (ORF). Sequence alignment was performed between serpin amino acid sequences of the migratory locust (Data S2) and other serpins sequences of silkworm, *Bombyx mori*, and fruit fly, *Drosophila melanogaster*, published by Universal Protein Knowledgebase (UniProt). The neighbor‐joining (NJ) method was used to construct phylogenetic trees by mega 6.0 software (Molecular Evolutionary Genetics Analysis Version 6.0), and 1000 bootstrap tests were performed [25].

### Advanced structure analysis of serpin proteins

Using the Self‐Optimized Prediction method With Alignment (SOPMA) online server (https://npsa-prabi.ibcp.fr/cgi-bin/npsa_automat.pl?page=/NPSA/npsa_sopma.html), we analyzed the secondary structure of serpin proteins and predicted the tertiary structure of serpin proteins by expasy software (Swiss Institute of Bioinformatics).

### Clone of* serpin7* gene

**Fig. 1:**
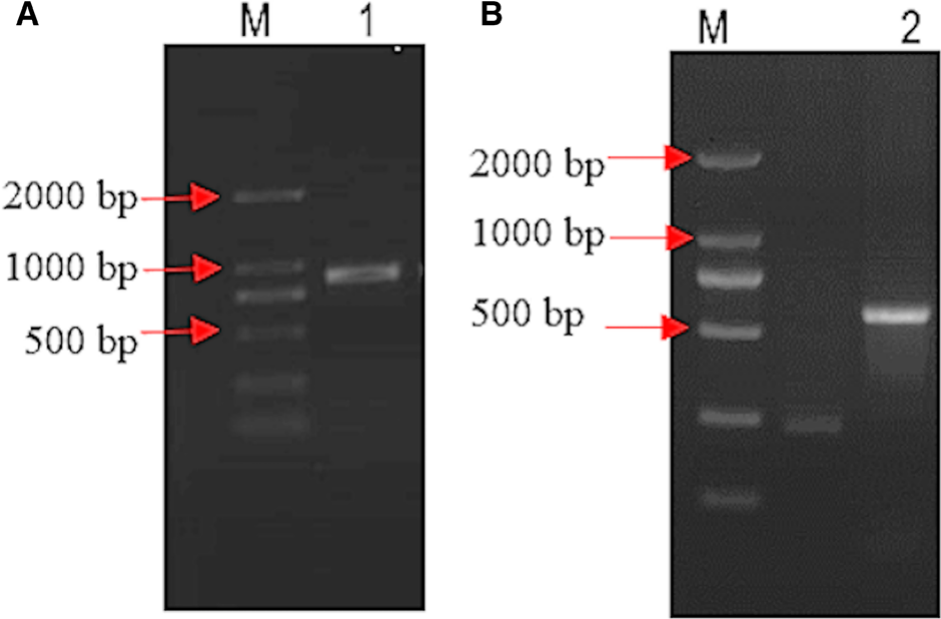
(A, B) Electrophoresis pattern of *serpin7* by PCR and *serpin7* dsRNA. 1, PCR fragment of *serpin7* gene; 2, *Serpin7* dsRNA; M, marker.

Third‐instar nymph of locusts was dissected, and the digestive tract was clinically removed. The remaining tissues were used for the extraction of total RNA. TRIcom Reagent (Tianmo, Huailai, China) was used to extract RNA. Total RNA was isolated according to the manufacturer’s protocol. The quality was checked on a spectrophotometer with *A*_260_/*A*_280_ between 1.9 and 2.0, whereas the reliability of RNA was confirmed on 1% agarose gel, which gave three clear bands. cDNA was synthesized according to the PrimeScript™ II 1st strand cDNA Synthesis Kit (TaKaRa, Dalian, China). By analyzing transcriptome of the migratory locust, we obtained the sequence of *serpin7* gene, and primers were then designed by dnaman software (version 7.212; Lynnon Corp.). Using cDNA of migratory locust as a template, we amplified the *serpin7* gene by primers *serpin7*‐1F/*serpin7*‐1R. The PCR‐amplified fragment was 894 bp (Fig. 1). The obtained PCR product was purified by TIANgel Midi Purification Kit (Tiangen, Beijing, China) and was connected to the 1‐μL pMD19‐T vector (TaKaRa, Dalian, China), 6 μL solution (TaKaRa, Japan) and 3 μL DNA to incubate at room temperate for 6 h. Later, the recombinant was transformed into *Escherichia coli* Trans1‐T1 strain, and 500 μL LB (lysogeny broth) liquid medium was added. Notably, no restriction enzymes were used. The obtained product was allowed to shake at 200 r.p.m. at 37 °C for 2 h. A total of 100 μL bacterial solution was applied to LB solid medium, including 1% of ampicillin. The medium was incubated at 37 °C for 12 h. The recombinant colonies were transferred into liquid LB culture medium containing 1% ampicillin and were shaken for 3–6 h at 37 °C. Finally, the medium for PCR template was prepared. Primers for this particular study were synthesized by Sangon Biotech Co. Ltd. (Beijing, China) (Table 1).

**Table 1:** List of specific primers used and synthesized for this study.

Primers	Primer sequences (5'‐3')	Intention
*Serpin7*‐1F	TTTCTCTCCAGCCAGCAT	Clone of *serpin7* gene
*Serpin7*‐1R	GCTTCAGTGCCTTCCTCAT	
*Serpin7*‐2F	TAATACGACTCACTATAGG CTGCTCAGGAGATTGTAGAGG	Synthesis of *serpin7* dsRNA
*Serpin7*‐2R	TAATACGACTCACTATAGG GACCATCATTCTTCTTTGGC	
*Serpin7*‐3F	GAAGGGAACTGATGACACGG	*Serpin7* primer for RT‐qPCR
*Serpin7*‐3R	TCTTGCTCTCAACCCATTCA	
*Easter*‐F	CGCATCGGATACATCGGGT	*Easter* primer for RT‐qPCR
*Easter*‐R	TTCTTGAAGGCGGGCTTG	
*Toll*‐F	GGCTGTAATGAATGGGGAA	*Toll* primer for RT‐qPCR
*Toll*‐R	GTAAACTGGAACTGGTGCG	
*Pelle*‐F	CCAACACGGAGAATAGATAGT	*Pelle* primer for RT‐qPCR
*Pelle*‐R	TGGTAAAATTCCAAGGTAGA	
*MyD88*‐F	GGCTTCCTCCTCAGCATCT	*MyD88* primer for RT‐qPCR
*MyD88*‐R	GACCTCCAACCAAATCACG	
*Cactus*‐F	CAGCGGTGCTTGCCTCTAC	*Cactus* primer for RT‐qPCR
*Cactus*‐R	TTTTCCTCCAACCTGTCCT	
*Actin*‐F	GTTACAAACTGGGACGACAT	Reference gene of RT‐qPCR
*Actin*‐R	AGAAAGCACAGCCTGAATAG	

### Synthesis and injection of double‐stranded RNA of *serpin7* gene

Recombinant plasmid including *serpin7* gene fragment was extracted by using EZgene™ Plasmid Miniprep Kit (Biomiga, San Diego, CA, USA). Using the recombinant plasmid as template, the *serpin7* gene was amplified by primers *serpin7*‐2F/*serpin7*‐2R (Table 1). The amplified PCR products were then purified with TIANgel Midi Purification Kit (Tiangen), followed by quantification through NanoPhotometer™ (Implen GmbH, Munchen, Germany). *Serpin7* double‐stranded RNA (dsRNA) was synthesized using the T7 RiboMAX™ Express RNAi System Kit (Promega, Madison, WI, USA). The expected size of *serpin7*’s dsRNA was 602 bp (Fig. 1). dsRNA concentration of *serpin7* was detected by a NanoPhotometer™ (Implen, GmbH, München, Germany), and the final concentration was adjusted to 1 μg·μL^−1^ for further analysis.

Twenty‐five female *L. migratoria* were selected from each photoperiod within 24 h after adults were injected with 10 μL *serpin7* dsRNA (μg·μL^−1^) between the third and fourth abdominal segments. Double‐distilled water (ddH_2_O) as control was injected in a similar manner to the selected females. Dissecting the whole bodies of dsRNA‐injected and control group’s adult locusts after 36 h, we obtained hind leg, ovary and fat body. The efficiency of RNAi‐mediated knockdown was determined with real‐time quantitative PCR (RT‐qPCR).

### RT‐qPCR

To check the efficiency of RNAi‐mediated knockdown in different tissues of *L. migratoria*, we dissected out the hind leg, ovary and fat body from each treatment. RNA was extracted from each sample using TRIcom Reagent (Tianmo) followed by estimating the RNA concentration through a NanoPhotometer™ (Implen, GmbH). For reverse transcription, 5 μL of total RNA was reverse transcribed with PrimeScript™ II 1st strand cDNA Synthesis Kit (TaKaRa, Dalian, China). To evaluate RNAi efficiency, we used primers *serpin7*‐3F/*serpin7*‐3R to amplify endogenous *serpin7* gene on the ABI 7500 Real‐Time PCR System (Applied Biosystems, Foster City, CA, USA). RT‐qPCR was performed with the Bester^®^ SYBR Green qPCR MasterMix (DBI^®^ Bioscience, Berlin, Germany). *β‐actin* was used as a reference gene in the study. A total of three technical replicates were set up. The relative mRNA level was calculated by $2^{-\Delta \Delta C_{t}}$ method [26], where

$$\Delta \Delta C_{t}=\left[\left(C_{t}\left(\text{Target}\right)-C_{t}\left(\text{actin}\right)\right)\text{Treatment}-\left(C_{t}\left(\text{Target}\right)-C_{t}\left(\text{actin}\right)\right)\text{control}\right].$$

### ELISA

PPO activity was detected using Insect PPO ELISA Kit (Collodi Biotechnology Co., Ltd., Quanzhou, China) according to the manufacturer’s protocol. The standard curve was generated by plotting the average absorbance (*A*_450_ nm) obtained for each of the six standard concentrations on the vertical (*x*) axis versus the corresponding concentration on the horizontal (*y*) axis. First, we calculated the mean *A*_450_ value for each standard and sample. Later, all of the *A*_450_ values were subtracted by the mean value of the blank well before interpretation of results. We constructed the standard curve using graph paper or statistical software. The *A*_450_ value of the sample was substituted into the equation, and the concentration of the sample was then calculated.

### Diapause rate

Remaining locusts were reared at 28 °C, until eggs laying. The numbers of hatched nymph of locusts (*D*_1_) and the unhatched eggs (*D*_2_) were counted, and the diapause rate (%) was calculated accordingly:

$$\text{Diapause}\;\text{rate}\left(\%\right)=\frac{D_{2}}{\left(D_{1}+D_{2}\right)}\times 100.$$

### Statistical analysis

Independent samples *t*‐test was used for measuring mRNA levels, enzyme activities and diapause rate. Statistically significant differences were considered on an error probability of *P* < 0.05. Data are presented as means ± SE. Asterisks on the bars in the figures represent significant differences among the treatments and control. Statistical analyses were performed using spss software version 16.0 (SPSS Inc., Chicago, IL, USA), whereas graphpad prism software version 6.01 (GraphPad Software Inc., San Diego, CA, USA) was used for constructing the graphs.

## Results

### Identification of the serpin genes

Seven nonredundant serpin protein sequences were identified by transcriptome sequencing of diapause and nondiapause eggs in the migratory locust and were respectively named from *serpin1* to *serpin7* (Table 2). The encoding gene of serpin proteins ranged from 987 bp (serpin1) to 1500 bp (serpin5). The isoelectric point (pI) was between 5.13 (*serpin3*) and 8.36 (*serpin4*). Similarly, prediction of subcellular localization was performed by wolf psort software (http://wolfpsort.org/). Higher value means accurate prediction. After analysis, *serpin1* was mainly distributed in the endoplasmic reticulum, nucleus and cytoplasm followed by a small amount of distribution in mitochondria and peroxisome. Among them, four proteins were predicted to be endoplasmic reticulum localization, including serpin2, serpin3, serpin5 and serpin6. Moreover, two of the serpin proteins were predicted to be localized in cytoplasm, including serpin4 and serpin7 (Table 3).

**Table 2:** Characteristics and features of serpins in *L. migratoria*.

Protein name	ORF	Amino acids	Molecular formula	Molecular weight	Theoretical pI
Serpin1	987	328	C_1655_H_2623_N_445_O_492_S_9_	36.92	5.9
Serpin2	1164	387	C_1945_H_3078_N_490_O_559_S_18_	42.85	6.73
Serpin3	1155	384	C_1966_H_3056_N_494_O_569_S_13_	43.13	5.13
Serpin4	1176	391	C_1997_H_3117_N_519_O_565_S_14_	43.89	8.36
Serpin5	1500	499	C_2543_H_3960_N_664_O_739_S_8_	55.92	5.91
Serpin6	1446	481	C_2402_H_3794_N_640_O_728_S_11_	53.64	5.86
Serpin7	1161	386	C_1933_H_3106_N_494_O_567_S_20_	42.98	6.08

**Table 3:** Subcellular localization and prediction of serpins using wolf psort software.

Protein name	Plasma membrane	Endoplasmic reticulum	Nucleus	Cytoplasm	Mitochondria	Peroxisome	Lysosome	Secreted
Serpin1	–	8	9	6.5	3	2.5	–	2
Serpin2	1	16	3	–	–	4	–	2
Serpin3	2	10	4	5	1	–	–	6
Serpin4	4	5	–	14	2	5	–	2
Serpin5	7	11	–	–	–	1	2	11
Serpin6	7	12	–	–	–	1	3	9
Serpin7	–	–	–	22.5	8	–	–	–

### Structure and phylogenetic tree of serpin protein

**Fig. 2:**
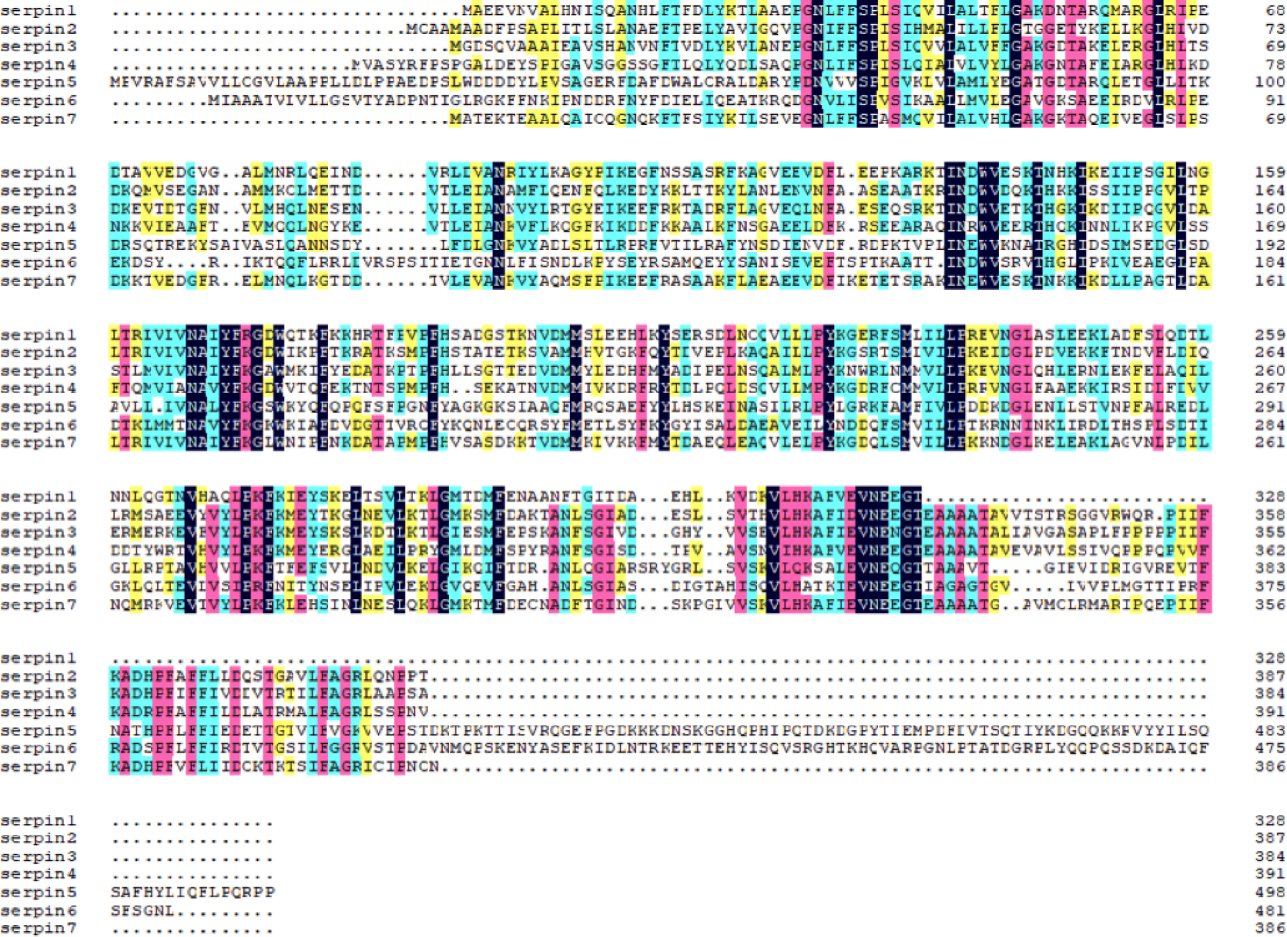
Homology of serpin proteins in *L. migratoria* constructed by using dnaman software.

**Fig. 3:**
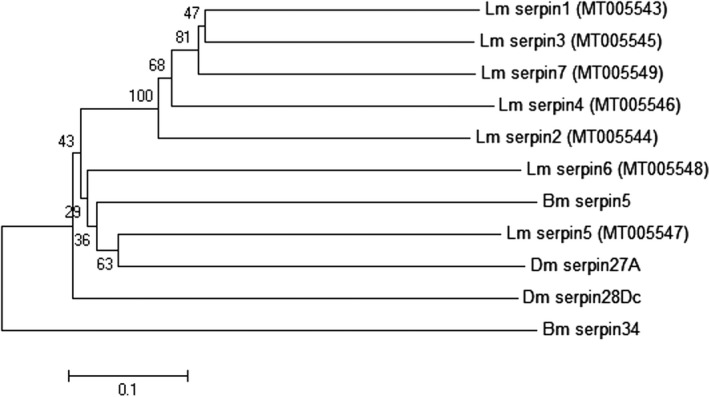
Phylogenetic tree of insect serpin proteins constructed by mega 6.0 and the NJ method. A species acronym is added before the name of each serpin protein, whereas Dm, Bm and Lm, respectively, represent *D. melanogaster*, *B. mori* and *L. migratoria*. GenBank accession numbers were provided for our nucleotide sequences, where *serpin1*, *serpin2*, *serpin3*, *serpin4*, *serpin5*, *serpin6* and *serpin7* are respectively numbered MT005543, MT005544, MT005545, MT005546, MT005547, MT005548 and MT005549.

Amino acid sequence alignment of seven serpin proteins was performed using dnaman software (version 7.212; Lynnon Corp.). High similarity among seven serpin proteins was found (Fig. 2). The phylogenetic tree was constructed by comparing the amino acid sequences of seven serpin proteins of migratory locust (Data S2) using the NJ method (Fig. 3). Two of the serpin proteins matched with fruit fly, *D. melanogaster*, and two with silkworm, *B. mori*. Through phylogenetic analysis, it was found that serpin5 in the migratory locust is closely related to *serpin27A* of *D. melanogaster*, whereas the relationship between *serpin1*, *serpin7*, *serpin3* and *serpin4* was relatively close; however, among them, the relationship between *serpin1* and *serpin3* was much closer. *Serpin1* was mainly distributed in the endoplasmic reticulum, nucleus and cytoplasm followed by a small amount of distribution in mitochondria and peroxisome predicted by wolf psort software (http://wolfpsort.org/).

### Analysis of the advanced structure of serpin proteins in the migratory locust

**Fig. 4:**
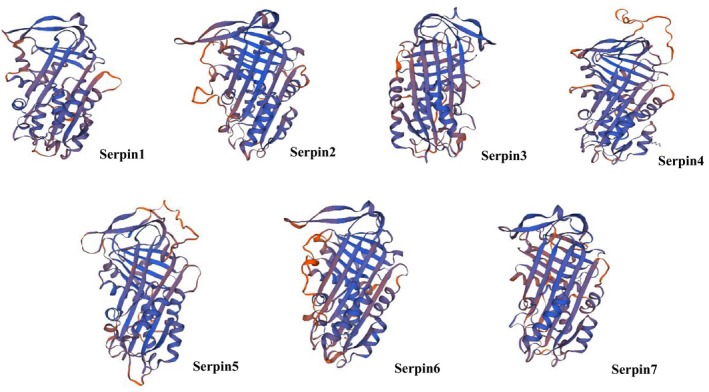
Tertiary structures of the serpin proteins obtained through expasy. Serpin1: α‐helix, 54.27%; extended strand, 10.37%; β‐turn, 6.40%; random coils, 28.96%. Serpin2: α‐helix, 45.99%; extended strand, 16.80%; β‐turn, 5.43%; random coils, 31.78%. Serpin3: α‐helix, 45.57%; extended strand, 15.36%; β‐turn, 6.25%; random coils, 32.81%. Serpin4: α‐helix, 44.50%; extended strand, 14.07%; β‐turn, 7.16%; random coils, 34.27%. Serpin5: α‐helix, 35.47%; extended strand, 16.23%; β‐turn, 6.01%; random coils, 42.28%. Serpin6: α‐helix, 34.79%; extended strand, 13.96%; β‐turn, 4.58%; random coils, 46.67%. Serpin7: α‐helix, 46.11%; extended strand, 16.32%; β‐turn, 5.96%; random coils, 31.61%.

The secondary and tertiary structures of the seven serpin proteins (serpin1–serpin7) were analyzed by the SOPMA online server and expasy software (Table 4; Fig. 4). There was a slight difference in the ratio of four secondary structures (α‐helix, extended strand, β‐turn and random coil) among the serpin proteins, in which the amino acids proportion of α‐helix was 34.79–54.27%, whereas β‐turn was 4.58–7.16%. Similarly, the amino acids proportion of extended chains was 10.37–16.80%. Moreover, the ratio of random coils ranged from 28.96% to 46.67%. Furthermore, secondary structures of α‐helix and random coils were dominant in serpin proteins. The tertiary structure of serpin was similar with three β‐turn and eight to nine α‐helices (Fig. 4).

**Table 4:** Secondary structure of the serpin proteins in *L. migratoria*.

	Number/Percentage			
	Random coil	α‐Helix	Extended strand	β‐Turn
Serpin1	101/30.79	168/51.22	39/11.89	20/6.10
Serpin2	131/33.85	175/45.22	60/15.50	21/5.43
Serpin3	128/33.33	164/42.71	69/17.97	23/5.99
Serpin4	138/35.29	182/46.55	56/14.32	15/3.84
Serpin5	217/43.49	177/35.47	75/15.03	30/6.01
Serpin6	215/44.70	180/37.42	67/13.93	19/3.95
Serpin7	138/35.75	170/44.04	59/15.28	19/4.92

### RNAi efficiency

**Fig. 5:**
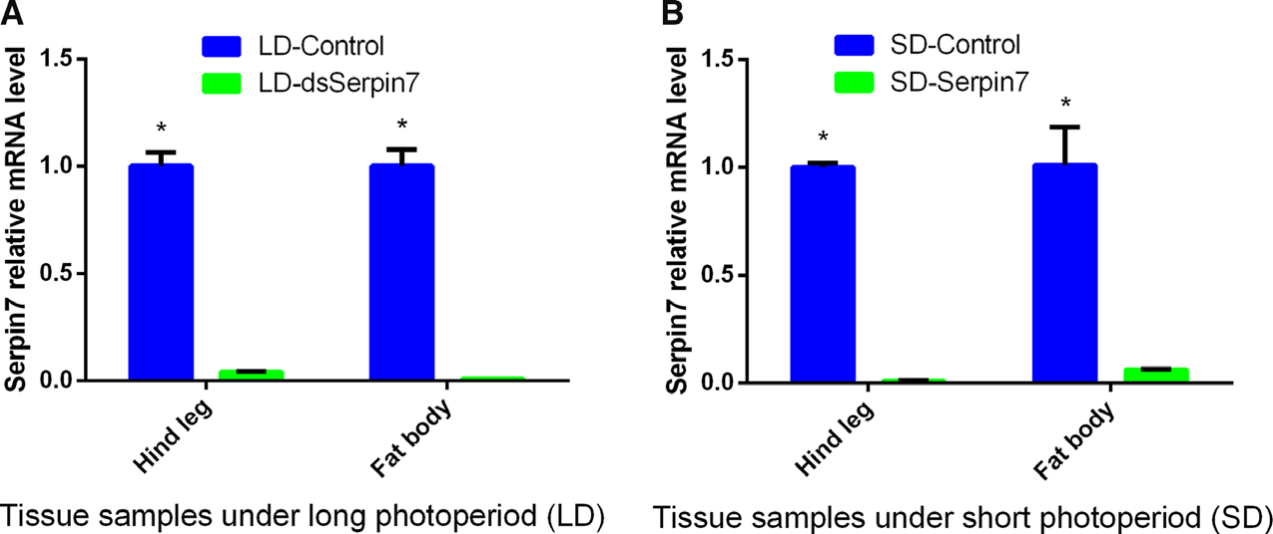
Relative mRNA levels of *serpin7* after RNAi under two photoperiodic regimens: (A) long photoperiod and (B) short photoperiod. Three replicates are used for each treatment. Asterisks (*) indicate a standard error probability of *P* < 0.05 by Student’s *t*‐test.

To verify the function of *serpin7* on regulating locust diapause, we synthesized and subsequently injected dsRNA of *serpin7* into female adults of *L. migratoria* to RNAi the *serpin7* under long and short photoperiods. RNAi efficiency of *serpin7* was confirmed through RT–qPCR. The results showed that the mRNA level of *serpin7* gene in hind leg and fat body was significantly different as compared with control (*P* < 0.05) under both long (relative humidity [RH] 60%; 30 °C; 16 h light:8 h darkness) and short (RH 60%; 27 °C; 10 h light:14 h darkness) photoperiods (Fig. 5). The results further confirmed that the *serpin7* gene was successfully interfered under two photoperiods. Under the long photoperiod, mRNA level of *serpin7* gene in the hind leg decreased by 95.98% compared with that of the control, whereas mRNA level of *serpin7* gene in fat body decreased by 99.08%. Furthermore, under the short photoperiod, mRNA level of *serpin7* gene in hind leg decreased by 99.14% compared with that of the control, whereas mRNA level of *serpin7* gene in fat body decreased by 93.85%.

### Effects of* serpin7* RNAi on the Toll pathway

**Fig. 6:**
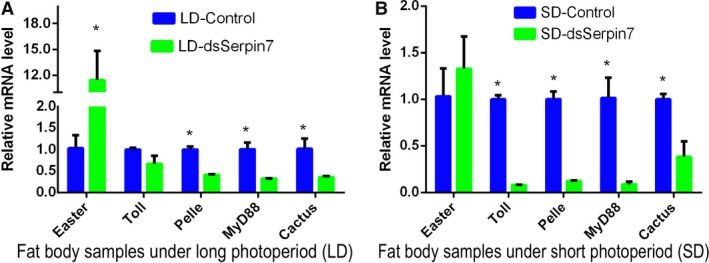
Data acquired from fat body samples and relative mRNA level of genes in the Toll pathway after *serpin7* RNAi under two photoperiodic regimens: (A) long photoperiod and (B) short photoperiod. Three replicates are used for each treatment. Asterisks (*) indicate a standard error (SE) probability of *P* < 0.05 by Student’s *t*‐test.

ds*serpin7* and ddH_2_O were injected into the female locusts within 24 h after adulthood under both long and short photoperiods. Locusts were dissected after 36 h to collect the fat body. The mRNA relative level of *Easter*, *Toll*, *Pelle*,* MyD88* and *Cactus* genes in the Toll pathway was checked (Fig. 6). Under the long photoperiod, the mRNA level of *Easter* in fat body was significantly up‐regulated (*P* < 0.05), whereas the mRNA levels of *Pelle*, *MyD88* and *Cactus* in fat body were significantly down‐regulated (*P* < 0.05). Similarly, the mRNA level of *Toll* gene in the treatment group was down‐regulated as compared with the control group, but with no significant difference. Under the short photoperiod, the variation trend of gene levels was similar to that of the long photoperiod. The mRNA relative levels of *Toll*, *Pelle*, *MyD88* and *Cactus* genes were significantly lower than those of the control group (*P* < 0.05), whereas the mRNA level of *Easter* gene was increased but did not reach a level of significant difference.

### Effect of RNAi‐mediated silencing of* serpin7* gene on activity of PPO

**Fig. 7:**
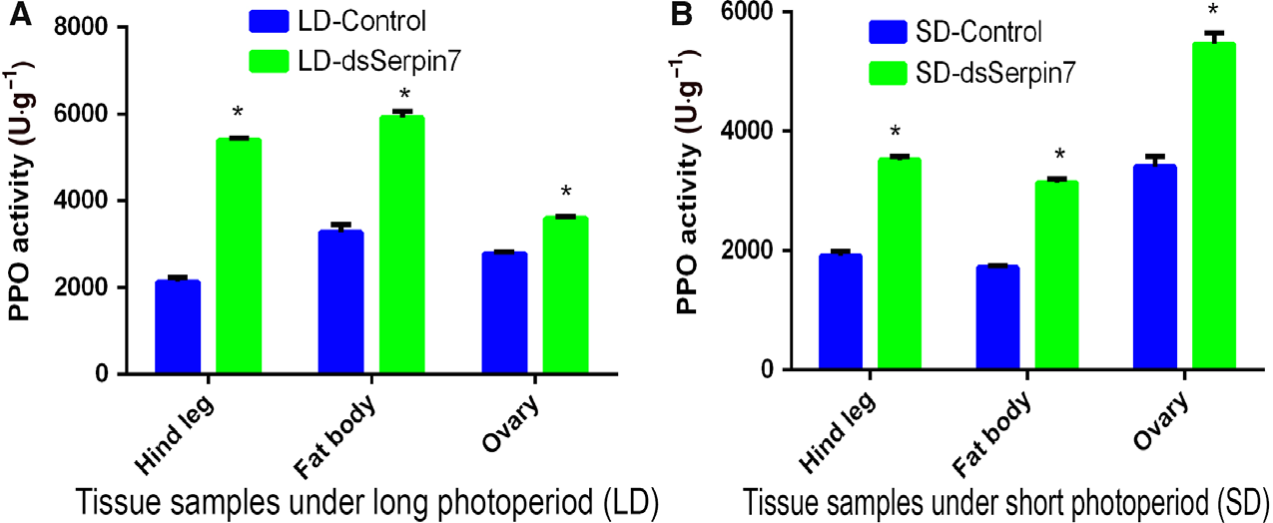
PPO activity in the migratory locust after *serpin7* RNAi under two photoperiodic regimens: (A) long photoperiod and (B) short photoperiod. Three replicates are used for each treatment. Asterisks (*) indicate a standard error probability of *P* < 0.05 by Student’s *t*‐test.

Under long and short photoperiods, the female adults were injected with dsRNA of *serpin7* and ddH_2_O, respectively. After 36 h, the female locusts were dissected to obtain the hind legs, fat bodies and ovaries. The PPO content was detected (Fig. 7). Results showed that the content of PPO in the hind leg, fat body and ovary significantly increased (*P* < 0.05) after *serpin7* RNAi under both long and short photoperiods.

### Diapause rate

**Fig. 8:**
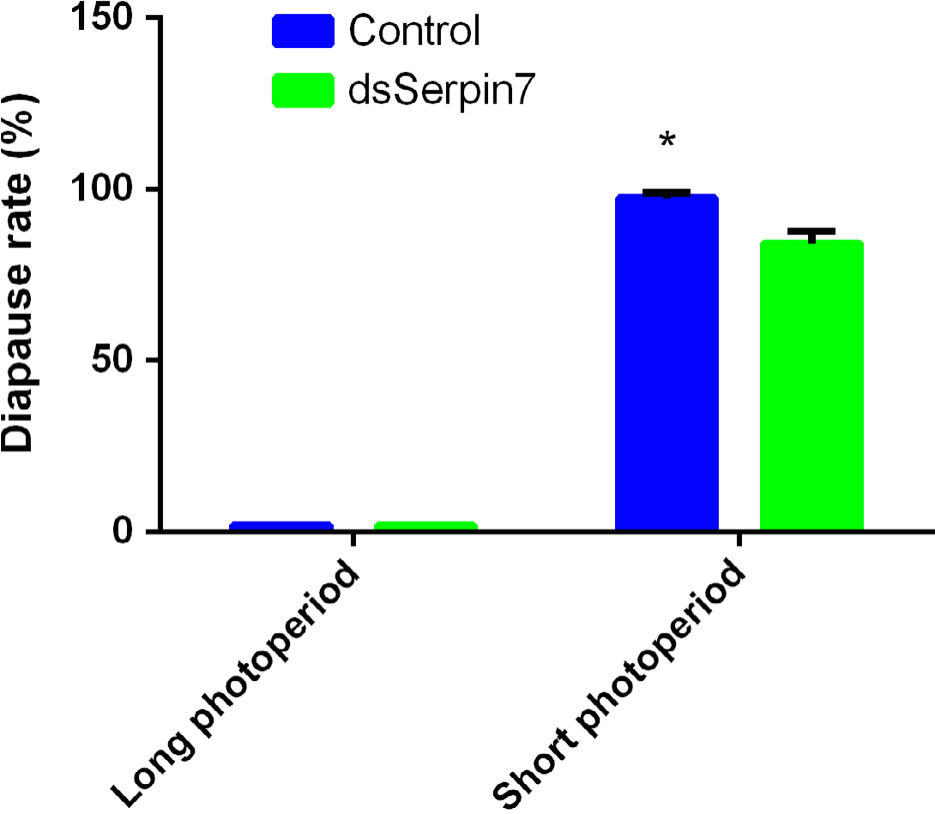
Diapause rate of migratory locusts by injecting ds*serpin7* along with control under both long and short photoperiods. Three replicates are used for each treatment. Asterisk (*) indicates a standard error probability of *P* < 0.05 by Student’s *t*‐test.

Under the long photoperiod, RNAi‐mediated silencing by injecting *serpin7* dsRNA had no effect on diapause rate. However, under the short photoperiod, diapause rate (%) of the individuals injected with *serpin7* dsRNA was significantly reduced (*P* < 0.05). Diapause rate in the dsserpin‐injected group was 84.19%, which was 13.25% lower than that of the control group. The result suggests that *serpin7* may play a modulatory role in egg diapause of the migratory locust (Fig. 8).

## Discussion

Serpins are a superfamily of proteases found in serine protease inhibitors, usually consisting of 350–400 amino acids [4]. At the C terminus of serpin, there is a reactive central ring exposed outside the main body [27]. Sequence of serpin is highly conserved, but its function is highly differentiated; that is why serpin has an irreversible inhibitory effect with a suicidal nature [7], just like trypsin inhibitors, which affect the growth, development and survival of insects [28]. Serpins play an important role in innate immunity of insects [19]. Currently, a negatively correlated serpin was found to be associated with the Toll signaling pathway of fruit flies [29, 30], and other serpins were mostly involved in melanization caused by PO cascade [31, 32]. In addition, a specific group of serpins is involved in tissue synthesis and embryonic development [17, 18, 20].

Serpins regulate some innate immune responses of insects by inhibiting endogenous protease [33]. In insects, serpins have been identified in several species, but extensive studies of insect serpins are mainly focused on fruit fly, *D. melanogaster*, and tobacco horn worm, *Manduca sexta*. So far, six *M. sexta* serpins have been characterized and shown to be inhibitory [13, 34, 35, 36, 37]. To investigate the effect of *serpin* gene on diapause of migratory locust, we subsequently cloned* serpin7* of the migratory locust in this particular study. The ORF of *serpin7* gene was 1161 bp, encoding 386 amino acid residues, and had the molecular formula of C_1933_H_3106_N_494_O_567_S_20_. The subcellular localization of *serpin7* was predicted to be cytoplasmic and mitochondrial. Meanwhile, the molecular mass of the protein was 42.98 kDa, which was similar to that of serpin’s superfamily protein [38]. The theoretical pI of serpin7 was 6.08, and there was no signal peptide. It belongs to the serpin superfamily and was a typical inhibitory serpin. Multiple sequence alignment showed that seven serpin amino acid sequences of the migratory locust showed high similarity (Fig. 2). Phylogenetic analysis was performed on serpin amino acid sequences by the NJ method, including seven serpin proteins in the migratory locust, two serpin proteins in fruit flies and two serpin proteins in silkworms. We observed a close relationship between serpin1 and serpin7 of migratory locusts. Moreover, serpin5 of migratory locusts was closely related to that of serpin27A of *Drosophila* [39, 40]. In addition, serpin6 of the migratory locust was closely related to serpin28Dc of *Drosophila* [41]. Furthermore, serpin1, serpin7, serpin3 and serpin4 were closely related with each other (Fig. 3).

Down‐regulation of the expression of specific genes through RNAi has been widely used in entomological research for functional genomics in a variety of insects, and its potential for RNAi‐based pest control has been increasingly emphasized mainly because of its high specificity [42, 43]. We used the RNAi technology to mediate silencing of *serpin7* gene of migratory locust for elaborating the effect of serpin on diapause of locust [23, 24, 44]. The RNAi efficiency of *serpin7* was checked by RT–qPCR, and the results revealed successful knocking down of *serpin7* gene. Our results showed the 100% hatching of all eggs under the long photoperiod, for both treated and untreated (control) groups with zero diapause. In contrast, under the short photoperiod, diapause rate of the individuals injected with *serpin7* dsRNA was significantly reduced as compared with the control group. Results suggested that maternal serpin7 promotes the egg diapause process of *L. migratoria*. Serpins are an important regulator in PO cascade reaction [32, 39, 45]. To further understand the regulatory mechanism, we checked the effect of *serpin7* RNAi on PPO activity in *L. migratoria*. Results showed that PPO activity increased significantly in the hind leg, fat body and ovary after *serpin7* RNAi. PO plays an important role in melanization [31, 32] and synthesis of antimicrobial peptides, where the proPO mainly exists as an inactive precursor [46, 47]. proPO regulates various downstream factors, such as protease, protease inhibitors in *Drosophila* and *Mandu casexta* [48, 49]. We speculated that serpin7 negatively regulates PPO and affects diapause of migratory locust eggs by PO cascade reaction. In addition, we examined the effect of *serpin7* gene RNAi on Toll pathway gene of the migratory locust. The results showed that after *serpin7* gene RNAi under long and short photoperiods, the mRNA level of *Easter* increased in fat body, whereas mRNA levels of *Toll*, *Pelle*, *MyD88* and *Cactus* genes decreased compared with the control group in fat body.

## Conclusions

RNAi of *serpin7* affected PPO activities in fat, hind leg and ovary of *L. migratoria* that ultimately revealed the possible role of serpin7 in locust diapause. Serpin7 may also be involved in the cascade reaction of the Toll signaling pathway, which needs to be further verified.

## Conflict of interest

The authors declare no conflict of interest.

## Author contributions

JC, DC and HU conceptualized the study. KH performed formal analysis. JC, DC and KH were involved in the investigation. KH and DC contributed to the methodology of the study. XT and ZZ were involved in project administration. DC and HU wrote the original draft. KH and HU contributed to the writing, reviewing and editing of the manuscript.

## Supporting information


**Data S1. **Selected serpins nucleic acid sequences.

**Data S2. **Selected serpins amino acid sequences.

## Data Availability

The sequences of *Serpin1*, *Serpin2*, *Serpin3*, *Serpin4*, *Serpin5*, *Serpin6* and *Serpin7* were deposited in GenBank with the accession numbers MT005543, MT005544, MT005545, MT005546, MT005547, MT005548 and MT005549, respectively.
