# Supplementary material for: Serpin7 controls egg diapause of migratory locust (Locusta migratoria) by regulating polyphenol oxidase
Source: FEBS Open Bio. 2020 Mar 24;10(5):707–17. doi: 10.1002/2211-5463.12825 (PMC7193170; doi:10.1002/2211-5463.12825)
Supplement: Supplementary file 1 — Data S1. Selected serpins nucleic acid sequences. [file FEB4-10-707-s001.docx]

**Data S1. Selected Serpins nucleic acid sequences.**

>Lm_serpin1

ATGGCAGAGGAAGTGAATCTAGCATTGCATAATATATCGCAAGCAAATCATCTCTTTACTTTCGACTTGTACAAGACTTTGGCAGCGGAGCCTGGGAACTTGTTCTTCTCTCCGTTGAGTATACAAGTAATTCTGGCGCTCACGTTTCTTGGAGCGAAAGACAATACGGCCAGGCAGATGGCCAAAGGACTGCGCATACCAGAGGACACAGCTGTCGTCGAGGATGGTGTCGGCGCTTTAATGAACAGATTACAGGAAATCAACGACGTGCGGCTTGATGTAGCCAACAGGATATATCTGAAAGCTGGATATCCCATCAAGGAAGGTTTTAATTCATCAGCATCTAGATTCAAGGCTGGAGTAGAGGAAGTAGATTTCCTAGAAGAACCGAAAGCGAGAAAAACCATAAATGACTGGGTAGAAAGCAAGACAAATCATAAGATAAAGGAAATAATTCCATCTGGTATATTGAATGGCTTAACTCGATTGGTGTTGGTCAATGCTATTTACTTCAGAGGCGACTGGCAGACAAAGTTTAAAAAGCATAGAACGTTTCCAGTGCCTTTCCACTCAGCTGACGGATCAACGAAGAATGTTGACATGATGTCTCTCGAGGAACACTTAAAGTACAGCGAGAGAAGTGATTTGAATTGCCAAGTCCTTCTCCTTCCTTATAAGGGAGAGAGGTTCAGCATGCTTATTTTACTACCCAGAGAGGTAAACGGATTGGCAAGTCTTGAGGAAAAACTTGCCGACTTCAGTCTTCAAGATACTCTTAACAACCTGCAAGGAACAAATGTACACGCACAATTACCAAAATTTAAAATTGAATACTCAAAAGAACTGACGAGTGTGCTAACAAAGCTCGGAATGACAGACATGTTTGAAAACGCTGCTAATTTCACTGGCATTACTGACGCAGAGCATCTGAAGGTGGACAAAGTCCTACATAAGGCTTTCGTTGAAGTCAACGAGGAGGGAACGGAAGCTGCTGCTGCTACTGCTGTGGTTGGCGTTCCATACTCGCTAACAATTTGGAAG

>Lm_serpin2

ATGGCTGCGGACTTTCCGTCAGCCCCTCTGATAACACTGTCCCTGGCAAACGCAGAGTTCACGCCAGAACTATATGCGGTAATAGGACAAGTACCAGGAAACATATTCTTCTCTCCGATCAGTATCCATATGGCTCTTATTCTGTTGTTTCTTGGGACTGGAGGTGAAACGTATAAAGAGCTTTTGAAAGGCCTTCACTTGGTCGATGACAAACAAATGGTATCGGAAGGAGCCAATGCAATGATGAAATGCCTTATGGAAACTACAGACGTGACACTAGAAATTGCAAACGCGATGTTTTTGCAAGAAAATTTTCAGCTGAAAGAAGACTACAAGAAGTTAACGACAAAGTATCTTGCAAACCTTGAAAACGTTAATTTCGCAGCGAGCGAAGCAGCGACGAAGAGAATAAACGATTGGGTAGATCAAAAAACCCATAAGAAGATAAGCAGTATTATACCGCCCGGTGTACTGACCCCTCTAACGCGCCTGGTGCTAGTGAATGCTATTTACTTCAAAGGTGATTGGATAAAACCATTCACCAAACGTGCTACTAAGTCTATGCCTTTCCACTCAACAGCGACAGAAACGAAGTCTGTTGCTATGATGCATGTTACAGGTAAATTCCAGTACACTGATGTGGAACCACTGAAGGCACAAGCCATCCTTCTTCCGTATAAGGGGTCACGGACTTCTATGTTAGTGATATTACCAAAGGAAATAGACGGGTTGCCAGATGTAGAGAAGAAATTCACTAACGATGTGTTTCTAGACATACAATTGAGAATGAGCGCAGAAGAAGTGTATGTATACCTACCGAAATTCAAGATGGAATATACAAAGGGCCTCAATGAAGTTCTAAAAACTCTCGGCATGAAGTCAATGTTCGATGCGAAGACAGCCAATCTGAGTGGCATCGCCGACGAGTCGCTGTCGGTGACGCACGTTCTGCACAAGGCGTTCATAGACGTCAACGAGGAGGGGACGGAGGCGGCCGCCGCCACAGCTGTCGTGACGTCCACAAGGTCTGGCGGGGTGCGCTGGCAGCGGCCGATAATCTTCAAGGCCGACCACCCGTTCGCGTTCTTCCTGCTGGACCAAAGTACCGGCGCCGTCCTCTTCGCTGGTCGCTTGCAGAATCCCCCAACGTAA

>Lm_serpin3

ATGGGTGACTCACAGGTCGCCGCCGCTATCGAGGCGGTATCGCACGCAAACGTCAACTTCACTGTGGACCTGTACAAGGTATTGGCGAATGAACCGGGGAATCTATTTTTCTCGCCACTCAGCATTCAGGTAGTTCTCGCTCTGGTGTTTTTCGGAGCAAAAGGTGACACAGCCAAGGAGTTGGAGCGAGGTTTGCACCTTACCAGCGACAAGGAAGTCACAGACACTGGCTTTAATGTTCTTATGCATCAGCTAAATGAGTCTGAGAATGTATTGCTTGAGATAGCGAACAATGTATACCTTCGGACTGGTTACGAAATCAAGGAGGAATTCAGAAAAACTGCTGATAGGTTCCTTGCTGGAGTTGAACAGCTCAACTTTGCTGAATCGGAACAGTCCAGAAAAACAATTAATGATTGGGTTGAAACAAAAACCCATGGGAAAATTAAGGATATAATTCCACAAGGAGTTTTGGATGCCAGCACTCTAATGGTGCTTGTCAACGCTATTTACTTCAAGGGCGCATGGATGAAGATATTTTACGAGGATGCAACCAAGCCCACGCCGTTTCATTTACTGTCAGGGACGACGGAAGATGTTGACATGATGTATCTGGAAGACCATTTTATGTATGCTGATATACCTGAATTAAATTCGCAGGCGCTTATGCTTCCTTACAAGAACTGGAGACTGAATATGATGGTGTTGCTACCAAAAGAAGTGAATGGTTTACAACACCTCGAGAGAAATCTTGAAAAATTTGAACTTGCGCAAATTCTGGAAAGAATGGAGAGAAAAGAAGTTTTCGTTTATTTACCAAAATTCAAAATGGAATACTCCAAGTCTTTAAAAGACACTCTGAAAACGCTCGGGATTGAGTCCATGTTCGAGCCTTCGAAAGCCAACTTCTCTGGCATTGTTGATGGGCATTATGTGGTAAGTGAGGTGTTGCACAAAGCATTTATAGAAGTCAACGAAAACGGCACCGAAGCAGCTGCCGCAACAGCCCTCATAGCGGTTGGGGCGAGCGCCCCTCTGTTTCCTCCGCCGCCGCCCATCATCTTCAAGGCCGACCACCCCTTCATATTCTTCATTGTGGATGACGTCACCCGCACCATCCTGTTCGCTGGACGTCTTGCGGCACCTTCTGCGTAATAACTGCGATT

>Lm_serpin4

ATGGTGGCGAGCTACAGATTTCCTTCGCCTGGAGCGCTGGACGAATATTCTCCTATTGGAGCTGTATCCGGGGGGAGCAGCGGTTTTACACTCCAACTATATCAAGACTTATCAGCACAGCCAGGAAACCTAATCTTCTCGCCCATCAGCCTACAGATAGCCCTGGTACTGGTGTATCTCGGGGCGAAAGGAAATACAGCGTTCGAGATTGCGAGGGGACTGCATTTAAAAGATAACAAGAAAGTCATCGAGGCTGCTTTCACTGAAGTAATGCAACAGCTGAATGGATATAAAGAGGTCACGCTAGAAATAGCGAACAAGGTTTTCTTAAAACAAGGATTTAAAATCAAAGACGATTTTAAGAAAGCGGCTCTTAAATTCAACTCTGGCGCTGAGGAATTGGATTTCAAACGCTCTGAAGAGGCAAGAGCTCAGATTAACAGATGGGTGGAAGAGAGAACGCACCAGAAGATAAATAACTTAATCAAGCCAGGTGTTTTGTCTTCGTTTACTCAGATGGTCATAGCAAACGCAGTATACTTCAAAGGAGACTGGGTAACACAGTTCGAGAAAACCAATACTTCTCCGATGCCGTTCCACTCGGAAAAAGCGACAAATGTGGACATGATGATAGTAAAGGACAGATTCAGGTACACTGATTTGCCTCAACTGGATTCCCAGGTTCTCCTGATGCCTTACAAGGGTGATCGCTTCTGTATGATGGTCCTGCTGCCAAGGAGAGTTAATGGTTTGTTCGCTGCTGAGAAGAAGATAAGGAGTATAGACCTGTTCGACGTTGTAGATGACACATATTGGCGCACAGTGCATGTATATCTGCCAAAGTTCAAAATGGAGTACGAAAGAGGACTCGCAGAAATATTACCACGTTATGGCATGCTCGACATGTTCAGTCCGTACAGGGCTAACTTCAGTGGAATCAGTGACACACCAGTTGCAGTTAGCAACGTCATCCACAAGGCGTTCGTTGAAGTCAACGAGGAAGGGACGGAGGCCGCGGCGGCCACTGCTGTTGAGGTCGCGGTGCTCTCCTCGATTGTGCAGCCGCCCCCGCAGCCCGTCGTCTTCAAAGCCGACCGGCCGTTCGCCTTCTTCATCCTGGATCTGGCGACGAGGATGGCGCTCTTCGCAGGACGCCTCTCTTCTCCGAATGTGTGATTA

>Lm_serpin5

ATCAGTCCGCGCCTTCTCAGCTGTCGTCCTCCTGTGCGGGGTGCTGGCCGCGCCGCCGCTGCTGGACTTGCCGCCTGCTGAGGACCCCAGCCTGTGGGACGACGACGACTACCTCCCCGTGTCTGCAGGCGAGCGGTTCGATGCCTTCGACTGGGCGCTTTGCCGGGCGCTCGATGCGCGCTACCCGGACAACGTGGTGGTTTCTCCCATCGGCGTCAAGCTGGTCCTAGCAATGCTGTACGAGGGCGCGACGGGAGACACCGCGCGCCAGCTCGAAACGGGGCTGCTGCTCACCAAGGACCGCTCGCAGACGCGCGAGAAGTACAGTGCCATCGTGGCGTCGCTGCAGGCAAACAACTCGGACTATCTGTTCGACTTGGGGAACAAAGTATACGCAGACTTGTCACTGACTCTACGCCCACGCTTTGTTACGATCCTGAGGGCGTTTTATAACAGTGATATTGAAAATGTCGACTTTAGAGATCCCAAAACTGTGCCCTTAATTAATGAATGGGTAAAAAATGCAACACGTGGTCACATTGACTCCATAATGAGTGAAGATGGCTTGTCGGACGCCGTCCTCCTGCTTGTGAACGCACTGTACTTCAAAGGCTCATGGAAATATCAATTTCAGCCTCAGTTCTCCTTTCCCGGCAATTTCTACGCAGGCAAAGGAAAGAGTATAGCCGCACAGTTCATGCGTCAGAGTGCAGAATTTTACTACTTGCACTCAAAGGAAATTAATGCAAGCATATTGCGACTACCGTATCTTGGCAGGAAATTTGCGATGTTTATCGTCCTGCCAGATGACAAGGATGGACTTGAAAATCTTTTGAGCACCGTAAACCCATTCGCTCTGCGAGAAGATCTCGGACTTCTCAGACCGACTGCAGTACATGTGGTGCTGCCAAAATTTACTTTCGAATTTTCTGTGCTCTTAAATGATGTTCTGAAGGAGCTGGGAATAAAGCAGATTTTCACTGACAGAGCTAATCTGCAAGGAATCGCGAGGAGTCGTTATGGAAGACTGTCAGTCTCGAAGGTTTTACAGAAATCAGCTCTAGAAGTAAACGAACAAGGAACTACAGCAGCGGCAGTTACAGGCATCGAGGTCATTGATCGAATTGGAGTCAGAGAGGTGACGTTCAATGCTACACATCCATTCCTTTTCTTTATCGAAGACGAGACGACCGGTACGGTAATATTCGTAGGAAAAGTTGTGGAACCATCAACTGACAAAACACCGAAAACTACGATTTCTGTTCGGCAAGGAGAGTTCCCGGGTGACAAGAAGAAAGATAACTCCAAAGGTGGTCATCAACCTCACATTCCTCAAACTGACAAGGACGGACCTTACACAATAGAAATGCCAGATTTTGACGTCACATCTCAAACAATCTACAAGGACGGACAGCAGAAAAAGCGTGTCTATTATATCTTATCCCAAAGCGCGTTTCATTACTTGATTCAATTTTTACCACAAAGGCCTCCGCAATAG

>Lm_serpin6

ATGATCGCCGCTGCCACAGTCTTGGTACTGCTGGGATCTGTTACATATGCAGATCCAAATACTATTGGACTAAGAGGAAAATTTTTCAACAAAATACCTAACGATGACAGATTCAATTATTTTGACATTGAATTAATACAGGAAGCCACCAAACGGCAGGATGGAAATGTATTGATTTCTCCAGTGAGTATCAAAGCTGCTCTCTTAATGGTACTTGAAGGTGCTGTAGGTAAATCTGCAGAGGAAATTAGAGATGTTCTGCGGTTGCCTGAAGAGAAGGATTCATATCGGATAAAAACACAGCAGTTCCTACGCAGACTTGATGTCAGATCTCCATCCATCACCATTGAGACAGGAAACAACCTCTTCATTTCAAATGACTTAAAGCCATATTCAGAATACAGATCTGCAATGCAAGAATATTATTCTGCTAATATCAGTGAAGTGGAATTCACTTCACCAACAAAAGCTGCAACAACAATCAATGACTGGGTCAGTAGAGTCACCCACGGACTTATACCAAAACTTGTTGAGGCAGAGGGTCTTCCAGCTGACACAAAGCTCATGATGACCAATGCAGTCTACTTCAAGGGAAAATGGAAAATAGCCTTTGATGTGGATGGTACAACAGTGAGATGTTTCTACAAACAAAATCTTGAATGCCAAAGATCATATTTTATGGAAACCCTGAGTTACTTCAAATATGGTTACATAAGTGCTCTTGATGCTGAGGCAGTGGAAATTCTTTATAATGATGATCAATTTTCTATGGTGATTCTACTTCCTACCAAGAGGAACAACATTAACAAATTAATCAGAGACTTAACACACAGCCCTCTTTCAGACACAATTGGAAAACTTCAGCTAACAGAAGTACTGGTGTCAATACCTCGTTTCAACATAACCTACAACTCTGAGCTCATACCTGTTTTAGAGAAGTTAGGAGTACAGGAGGTGTTTGGAGCTCACGCCAATCTCTCTGGTATTGCAAGTGACATAGGAACAGCTCATATCAGCCAAGTACTACATGCAACGAAGATTGAAGTTAATGAGGAAGGTACAATTGCTGGGGCTGGTACAGGTGTTCTGGTGGTTCCTCTAATGGGAACTACCATCCCAAGATTCAGAGCTGATAGTCCATTCTTATTTTTTATCCGTGATACTGTGACTGGAAGTATACTATTTGGGGGTAGAGTCTCAACGCCAGATGCAGTAAATATGCAACCGAGTAAAGAAAATTATGCATCAGAGTTCAAAATTGACTTGAATACTAGGAAAGAAGAGACAACAGAACATTATATTTCACAGGTATCACGAGGACATACAAAACATCAAGTAGCAAGACCAGGCAACCTTCCAACTGCAACCGATGGAAGACCATTATACCAACAACCACAGTCTAGTGATAAAGATGCTATTCAGTTCTCTTTTTCAGGAAATTTATAG

>Lm_serpin7

ATGGCAACTGAAAAAACAGAAGCAGCTCTCCAGGCAATATGTCAAGGAAACCAAAAATTTACATTCAGTATTTACAAGATTCTTTCAGAAGTGGAAGGTAACCTGTTTTTCTCTCCAGCCAGCATGCAAGTAATTTTAGCTCTTGTACATCTTGGTGCAAAGGGCAAAACTGCTCAGGAGATTGTAGAGGGTCTGAGTCTTCCATCTGATAAGAAGACTGTTGAGGATGGTTTCAGAGAATTGATGAATCAGCTGAAGGGAACTGATGACACGGTACTCGAAGTAGCAAACAAGGTTTATGCACAAATGAGTTTTCCAATCAAAGAAGAATTTAGAGCATCTGCTGCAAAATTTCTAGCTGAAGCTGAAGAAGTTGATTTTATCAAAGAAACAGAGACATCAAGGGCAAAAATCAATGAATGGGTTGAGAGCAAGACAAACAAGAAGATCAAAGATTTGCTTCCTGCAGGTACTTTGGATGCATTGACTCGTCTAGTGCTTGTAAATGCTATTTACTTCAAAGGCCTATGGAATATTCCATTCAATAAAGATGCCACAGCCCCCATGCCTTTCCATGTTAGTGCATCAGATAAGAAGACTGTTGATATGATGAAGCTAGTGAAAAAATTTATGTATACTGATGCAGAACAACTTGAAGCTCAAGTTCTTGAACTCCCTTACAAGGGAGACCAGTTAAGTATGGTGATCCTCTTGCCAAAGAAGAATGATGGTCTTAAGGAACTAGAAGCAAAGCTTGCTGGTGTGAATTTACCCGATATCTTGAACCAAATGCGTAAAGTTGAAGTGACTGTTTACTTACCCAAATTCAAATTGGAACACTCAATAAACCTGAATGAGAGTTTACAGAAACTGGGTATGAAGACTATGTTTGATGAGTGCAATGCGGATTTTACTGGTATCAATGACTCCAAACCTGGCCTTGTTGTTAGCAAAGTCTTGCACAAAGCATTCATAGAAGTAAATGAGGAAGGCACTGAAGCTGCAGCAGCAACTGGTGCAGTTATGTGCCTGAGGATGGCTCGAATTCCTCAAGAACCAATAATTTTCAAGGCAGATCACCCTTTTGTATTTTTAATTATTGATTGCAAAACAAAAACAAGCATTTTTGCTGGGCGCATCTGTATTCCAAATTGTAATTAGTGATAG
