# Supplementary material for: Serpin7 controls egg diapause of migratory locust (Locusta migratoria) by regulating polyphenol oxidase
Source: FEBS Open Bio. 2020 Mar 24;10(5):707–17. doi: 10.1002/2211-5463.12825 (PMC7193170; doi:10.1002/2211-5463.12825)
Supplement: Supplementary file 2 — Data S2. Selected serpins amino acid sequences. [file FEB4-10-707-s002.docx]

**Data S2. Selected Serpins Aminoacid Sequences**

>Lm_serpin1

MAEEVNVALHNISQANHLFTFDLYKTLAAEPGNLFFSPLSIQVILALTFLGAKDNTARQMARGLRIPEDTAVVEDGVGALMNRLQEINDVRLDVANRIYLKAGYPIKEGFNSSASRFKAGVEEVDFLEEPKARKTINDWVESKTNHKIKEIIPSGILNGLTRLVLVNAIYFRGDWQTKFKKHRTFPVPFHSADGSTKNVDMMSLEEHLKYSERSDLNCQVLLLPYKGERFSMLILLPREVNGLASLEEKLADFSLQDTLNNLQGTNVHAQLPKFKIEYSKELTSVLTKLGMTDMFENAANFTGITDAEHLKVDKVLHKAFVEVNEEGT

>Lm_serpin2

MCAAMAADFPSAPLITLSLANAEFTPELYAVIGQVPGNIFFSPISIHMALILLFLGTGGETYKELLKGLHLVDDKQMVSEGANAMMKCLMETTDVTLEIANAMFLQENFQLKEDYKKLTTKYLANLENVNFAASEAATKRINDWVDQKTHKKISSIIPPGVLTPLTRLVLVNAIYFKGDWIKPFTKRATKSMPFHSTATETKSVAMMHVTGKFQYTDVEPLKAQAILLPYKGSRTSMLVILPKEIDGLPDVEKKFTNDVFLDIQLRMSAEEVYVYLPKFKMEYTKGLNEVLKTLGMKSMFDAKTANLSGIADESLSVTHVLHKAFIDVNEEGTEAAAATAVVTSTRSGGVRWQRPIIFKADHPFAFFLLDQSTGAVLFAGRLQNPPT

>Lm_serpin3

MGDSQVAAAIEAVSHANVNFTVDLYKVLANEPGNLFFSPLSIQVVLALVFFGAKGDTAKELERGLHLTSDKEVTDTGFNVLMHQLNESENVLLEIANNVYLRTGYEIKEEFRKTADRFLAGVEQLNFAESEQSRKTINDWVETKTHGKIKDIIPQGVLDASTLMVLVNAIYFKGAWMKIFYEDATKPTPFHLLSGTTEDVDMMYLEDHFMYADIPELNSQALMLPYKNWRLNMMVLLPKEVNGLQHLERNLEKFELAQILERMERKEVFVYLPKFKMEYSKSLKDTLKTLGIESMFEPSKANFSGIVDGHYVVSEVLHKAFIEVNENGTEAAAATALIAVGASAPLFPPPPPIIFKADHPFIFFIVDDVTRTILFAGRLAAPSA

>Lm_serpin4

MVASYRFPSPGALDEYSPIGAVSGGSSGFTLQLYQDLSAQPGNLIFSPISLQIALVLVYLGAKGNTAFEIARGLHLKDNKKVIEAAFTEVMQQLNGYKEVTLEIANKVFLKQGFKIKDDFKKAALKFNSGAEELDFKRSEEARAQINRWVEERTHQKINNLIKPGVLSSFTQMVIANAVYFKGDWVTQFEKTNTSPMPFHSEKATNVDMMIVKDRFRYTDLPQLDSQVLLMPYKGDRFCMMVLLPRRVNGLFAAEKKIRSIDLFDVVDDTYWRTVHVYLPKFKMEYERGLAEILPRYGMLDMFSPYRANFSGISDTPVAVSNVIHKAFVEVNEEGTEAAAATAVEVAVLSSIVQPPPQPVVFKADRPFAFFILDLATRMALFAGRLSSPNV

>Lm_serpin5

MPVRAFSAVVLLCGVLAAPPLLDLPPAEDPSLWDDDDYLPVSAGERFDAFDWALCRALDARYPDNVVVSPIGVKLVLAMLYEGATGDTARQLETGLLLTKDRSQTREKYSAIVASLQANNSDYLFDLGNKVYADLSLTLRPRFVTILRAFYNSDIENVDFRDPKTVPLINEWVKNATRGHIDSIMSEDGLSDAVLLLVNALYFKGSWKYQFQPQFSFPGNFYAGKGKSIAAQFMRQSAEFYYLHSKEINASILRLPYLGRKFAMFIVLPDDKDGLENLLSTVNPFALREDLGLLRPTAVHVVLPKFTFEFSVLLNDVLKELGIKQIFTDRANLQGIARSRYGRLSVSKVLQKSALEVNEQGTTAAAVTGIEVIDRIGVREVTFNATHPFLFFIEDETTGTVIFVGKVVEPSTDKTPKTTISVRQGEFPGDKKKDNSKGGHQPHIPQTDKDGPYTIEMPDFDVTSQTIYKDGQQKKRVYYILSQSAFHYLIQFLPQRPPQ

> Lm_serpin6

MIAAATVLVLLGSVTYADPNTIGLRGKFFNKIPNDDRFNYFDIELIQEATKRQDGNVLISPVSIKAALLMVLEGAVGKSAEEIRDVLRLPEEKDSYRIKTQQFLRRLDVRSPSITIETGNNLFISNDLKPYSEYRSAMQEYYSANISEVEFTSPTKAATTINDWVSRVTHGLIPKLVEAEGLPADTKLMMTNAVYFKGKWKIAFDVDGTTVRCFYKQNLECQRSYFMETLSYFKYGYISALDAEAVEILYNDDQFSMVILLPTKRNNINKLIRDLTHSPLSDTIGKLQLTEVLVSIPRFNITYNSELIPVLEKLGVQEVFGAHANLSGIASDIGTAHISQVLHATKIEVNEEGTIAGAGTGVLVVPLMGTTIPRFRADSPFLFFIRDTVTGSILFGGRVSTPDAVNMQPSKENYASEFKIDLNTRKEETTEHYISQVSRGHTKHQVARPGNLPTATDGRPLYQQPQSSDKDAIQFSFSGNL

>Lm_serpin7

MATEKTEAALQAICQGNQKFTFSIYKILSEVEGNLFFSPASMQVILALVHLGAKGKTAQEIVEGLSLPSDKKTVEDGFRELMNQLKGTDDTVLEVANKVYAQMSFPIKEEFRASAAKFLAEAEEVDFIKETETSRAKINEWVESKTNKKIKDLLPAGTLDALTRLVLVNAIYFKGLWNIPFNKDATAPMPFHVSASDKKTVDMMKLVKKFMYTDAEQLEAQVLELPYKGDQLSMVILLPKKNDGLKELEAKLAGVNLPDILNQMRKVEVTVYLPKFKLEHSINLNESLQKLGMKTMFDECNADFTGINDSKPGLVVSKVLHKAFIEVNEEGTEAAAATGAVMCLRMARIPQEPIIFKADHPFVFLIIDCKTKTSIFAGRICIPNCN

>Dm_serpin27A

MTKMGGNLAVMLLSLFLSALATGNGNSIPTTTTPQGVFETRTDKLPGGAASVPSGAGIYDDIDTFVPFRSDSHDPFSWHLLKTVLQNETADKNVIISPFSVKLVLALLAEAAGAGTQTQVELANTQTDIRSQNNVREFYRKTLNSFKKENQLHETLSVRTKLFTDSFIETQQKFTATLKHFYDSEVEALDFTNPEAAADAINAWAANITQGRLQQLVAPDNVRSSVMLLTNLIYFNGLWRRQFATTFQGSFFRSKDDQSRAEFMEQTDYFYYTTSEKLKAQILRLPYKGKNSLFVLLPYALNGIHDLVKNLENDELKSAQWAMEEVKVKVTLPKFHFDYQQNLKETLRSLGVREIFEDSASLPGLTRGADVAGKVKVSNILQKAGINVNEKGTEAYAATVVEIENKFGGSTAIEEFNVNRPFVFFIEEESTGNILFAGKVHSPTTQN

>Dm_serpin28Dc

MWRLLLALLLVSSVCCESELFRDDLRTPETMAYINGLMQRRHQMQQEAQQHIQAIPPAVPLQSPGLVNGLGNQNDPALNRISGTSVKPSNLPAAYSNGYVDLATSDRIANSVLNFANILGQHLANGKTQIYSPLSIVHSLALLLLGAKGRSYEELSTVFDIPDTSRLHEQFGLMLQDLQQPTREAISAGRPLTDWRASSAMRSNRRAQRPGAHEVHLANGLFTQTGYTLNPDYRRVIVEVYASDLQIQDFEGSPATARYNINAYVAQHTKNHIENIIASDIPQTTRMILANALYFKAFWETDFIESATRPDNFYPNGEGTEPVMRVQMMATGGAYPYHEDHELGCKIIGLPYRGNLSTMYIIQPFKSSVRELMALQKRLTADKIESMISRMYRRAALVAFPKMHLTESVNLKTVMQRMGLGGIFSAVQNDLSLIATNEATRTNALGGNSLQNLEAQRRAGTGGARSDLVVDDIVHKVDFTVNEQGTEAAASSVTYLKKSGPDVLFRGDTPFMVLVRHDPTKLVLFYGLINEPPAAA

>Bm_serpin34

MRRSGRALSCAAAGLFCNLLYACIKLTESTDYGVPIEIKLWDTGRTPLAHMVDVTNEFGLKVLAEHNFLNENNIAFSPYGLMGILVALYEGVDGESSYQLQRAIQLPWNRKIMRVGFRDIHRTLKTYFVPEEGFLAGLALNNENVTFNENFKKILRFYGFDLDNDQLPALPNQTNKTMNETTPKSSTIATSQADGGDVSTGTTVTTENTREETTPATSRDTTANPNAETINTIATTTAIPSSAPNFESTTVGISTTTLQDIIKPLISSTETTTQIVTTTNSVTTSTNPTDSTTNENLSITTANPSSTDIDEETIAQQTSQATVALTQTENNEIDGTAAVTSSTVDLNFTESTTSVSTTEIQTDSVTFTTSTDKPLVTLSDTATSSDMTTAANGSSLETLERRKKSIVDFIFTNPPYVDDYLMYRSFDIPAEPPKPTFDEQMFLANGLKSVQVTYMHYDTILEHAYLPHLEASALRLPLDSERYYLLVVLPARGSAAELGRLLARMARESDLSDIYAALRPRRVKGIVPSFTVKGHVTLTTDLQKVSMI

>Bm_serpin5

MYSIAFVLFFVGACYCDVDFSERPRNFSIELLYHTQTQTDGHVVISPFGIWTLMTGIALGASGNSYRQLSRAFILPKNPATLIEGYKSLTDVVLDQTANGVSLVSRNFVFLDNDFTLLPAFRTTLQKDFGASIKVLDFSDPNSARIANTYIEKSGGRVSNVLRSDDFQESRLILTNVISFKGLWGLPFNKSDTELEPFYNEDRLVIGSVNMMYQKGQFPFSNLKKLKAFVLELPYGTDGKYSMLVILPHPRTKIADMYKNFADVNLKDVFKQLQKDVDDFGLEDVDVKLPRFQISTNVVLNKPLNDMGVYDIFQPDLANFQRITKENIFVSAIVHKADIEVTESGTVAFASTVATLTDRISAPAFHANRPFVYFVMEKTTTPVIFSGIYSKPTVY
